# Endophytic *Streptomyces* from honeybee hives inhibit plant and honeybee pathogens

**DOI:** 10.3389/fmicb.2025.1644842

**Published:** 2025-09-30

**Authors:** Claire Reichardt, David Estes, Caitlin M. Carlson, Cameron R. Currie, Daniel S. May

**Affiliations:** ^1^Department of Bacteriology, University of Wisconsin-Madison, Madison, WI, United States; ^2^Department of Chemistry, Washington College, Chestertown, MD, United States; ^3^Department of Biochemistry and Biomedical Sciences, McMaster University, Hamilton, ON, Canada

**Keywords:** endophyte, *Apis mellifera*, *Streptomyces*, natural products, *Paenibacillus larvae*, secondary metabolites, actinobacteria

## Abstract

Honey bees are the most common pollinator of crops worldwide. However, our reliance on honey bees to pollinate pesticide-treated monoculture crops, combined with their pest and disease susceptibility, have led honey bee populations to fluctuate in recent years. Current treatments for honey bee bacterial and fungal diseases are inadequate due to poor safety profiles and increased pathogen resistance to these treatments. There has been renewed interest in discovering natural products from actinobacteria associated with bees to use as new hive treatments; however, few studies have determined whether these microbes are truly unique to bees or part of their broader environment. We isolated actinobacteria from plant pollen and hive pollen stores and found that the isolated *Streptomyces* strains share many features with previously characterized endophytic *Streptomyces* strains. Selected *Streptomyces* strains were sequenced, and the genomes were used to search for phylogenetic relationships, identify genetic markers of endophytism, and compare biosynthetic gene clusters. LC-MS/MS was used to confirm the production and identities of the genetically predicted natural products. Finally, we tested the ability of the isolated actinobacteria to inhibit the growth of both plant and honey bee pathogens. Specific taxa, like *Streptomyces albidoflavus* and *Streptomyces olivaceus*, were regularly isolated from both plants and hives and produced many of the same natural products. These natural products and the *Streptomyces* strains that produce them may represent a starting point for antibiotics that could be used to help protect these critical pollinators.

## Introduction

The western honey bee (*Apis mellifera*) is the most common pollinator of crop plants in the world (Osterman et al., 2021). In the United States alone, pollinators are responsible for approximately $30 billion per year in crop pollination services (Khanna et al., 2021). This economic footprint does not include other products produced by bees that are used by humans such as honey, wax, and propolis. The beehive obtains most of their nutrition from the nectar and pollen of plants surrounding the hive. This close association with surrounding plants has become a burden, as bees are increasingly being used to pollinate large fields of monoculture crops with very little diversity in plant material and thus limited diversity of nutrients. This, along with increased pesticide use in agriculture and increased spread of pests and diseases, has led to fluctuating bee populations in recent years (Oddie and Dahle, 2024).

Honey bees live in dense hives of closely related individuals which makes them exceptionally susceptible to disease. Honey bees are host to a variety of parasites and viral, fungal, and bacterial pathogens. Many of these pathogens are opportunistic, such as the fungal pathogen *Aspergillus niger* and the bacterial pathogen *Serratia marcescens,* that are introduced to the hive through foraging behaviors (Raymann et al., 2018). Other pathogens, like *Paenibacillus larvae*, are specific to hives and can be transferred between hives by foraging bees or apiculturists. This Gram +, spore forming bacteria infects and kills the brood, larvae and pupae of the bees, diminishing the hive’s reproductive abilities and slowly killing it. The spore forming ability of *P. larvae* makes it particularly difficult to remove from the hive, as spores can remain dormant and persist on hive material or bee keeping tools before infecting new hives (Ebeling et al., 2016). Current treatments for *P. larvae* infection are incinerating the hive material or treating with one of two antibiotics, oxytetracycline or tylosin. Antibiotics can inhibit members of the bee gut microbiota and cause individual bees to starve (Raymann et al., 2017; Baffoni et al., 2021). Additionally, *P. larvae* has demonstrated increased levels of resistance to oxytetracycline, necessitating the discovery of new and safer antibiotics for apiculture (Kochansky et al., 2001; Evans, 2003).

Recent studies have sought to use bees and their hives as a source of bacteria capable of producing antibiotics and other natural products. Many of these studies have focused on *Streptomyces* spp. and other closely related actinobacteria due to their well-known ability to produce an outsized number of natural products compared to the size of their genomes (Chevrette and Currie, 2019). *Streptomyces* spp. have been documented in both the hive microbiota and on foraging bees in culture independent studies (Anderson et al., 2013; Corby-Harris et al., 2014). Other studies have used *Streptomyces* specific culturing techniques to isolate *Streptomyces* spp. from hives and bees. Grubbs et al. (2021) and Santos-Beneit et al. (2022) previously isolated *Streptomyces* spp. from hive material, specifically pollen stores, that produced known antibiotics. *Streptomyces* spp. have been found associated with other species of bees as well. Menegatti et al. (2020) studying native stingless bees (*Melipona scutellaris*), isolated *Streptomyces* from foraging bees and nurse bees that produced lobophorins and anthracycline antibiotics (Rodríguez-Hernández et al., 2019). Promnuan et al. (2021) identified *Streptomyces* from foraging black dwarf honeybees (*Apis adreniformis*) that inhibited several crop pathogens. While it has been suggested that these *Streptomyces* are uniquely associated with their respective bee species, the isolation of these bacteria from actively foraging bees and pollen stores in hives suggests that these inhibitory *Streptomyces* enter the hive through the plant foraging activities of bees.

*Streptomyces* spp. are conventionally isolated from soil and have long been recognized to be closely associated with the plant rhizosphere and plant health (Chen et al., 2016; Viaene et al., 2016; van der Meij et al., 2018; Vurukonda et al., 2018; Pronk et al., 2022; Wang et al., 2024). Several commercial biological fungicides, such as Actinovate™, contain *Streptomyces* strains, and are used to suppress plant fungal diseases (Crawford and Suh, 1993). *Streptomyces* spp. have also been isolated from within plant tissues as endophytes (Conti et al., 2016). Worsley et al. isolated five endophytic *Streptomyces* sp. strains from *A. thaliana.* They demonstrated the ability of some of the isolates to promote plant growth, produce plant hormones auxin and cytokinin, and produce natural products that inhibited plant pathogens. Additionally, they demonstrated that these endophytic *Streptomyces* could colonize plant tissues as vegetative mycelia through confocal microscopy experiments with eGFP-tagged *Streptomyces* (Worsley et al., 2020). This close association with plants further supports the possibility that *Streptomyces* spp. move via plant pollen from plants to bees during foraging activities. Kim et al. demonstrated this possibility by isolating the endophytic *Streptomyces badius* SP6C4 from strawberries. The endophyte inhibited both plant and bee pathogens and was also able to be transported between plants by foraging bees (Kim et al., 2019; Kim and Kwak, 2021).

A better understanding of the source of these hive-isolated beneficial bacteria will help in the discovery of antibiotics that can protect pollinators from pathogens. We hypothesize that many of these inhibitory *Streptomyces* isolated from hives and foraging bees are endophytic bacteria that enter hives via foraged pollen. By isolating actinobacteria from plant pollen and hive pollen stores, we demonstrate the isolated actinobacteria are taxonomically diverse, contain several genetic markers of endophytism, and are capable of producing similar known antibacterial and antifungal natural products that may be beneficial in both endophytic and hive environments.

## Materials and methods

### Plant pollen collection

Pollen was collected from plants in the Lakeshore Nature Preserve on the campus of University of Wisconsin-Madison in Madison, Wisconsin. The preserve consists of native Wisconsin plants growing in both prairie and temperate forest ecosystems. Pollen samples were collected at three time points, early April, early May, and mid-June, 2021. A diverse array of ten native Wisconsin plant species were selected for pollen sampling (Supplementary Figure 1). Five biological replicates were taken from each plant using sterilized forceps to remove the anthers, which were placed into sterilized Eppendorf tubes. The samples were refrigerated for less than 1 week until processed for bacterial isolation.

### Hive pollen collection

Hive pollen was collected from pollen stores of a honey bee hive maintained by the Currie Laboratory at the Microbial Sciences Building in Madison, Wisconsin, in early September, 2021. The hive is approximately 1.5 miles away from the Lakeshore Nature Preserve. Hive frames that contained pollen stores were sampled using a sterilized spade to remove the pollen. Ten pollen stores were sampled, and the pollen was placed in sterilized Eppendorf tubes and refrigerated for less than 1 week until processed for bacterial isolation.

### Pollen grain count estimation

A C-Chip disposable hemocytometer was used to estimate the number of pollen grains that were added to each isolation plate. This was done to eliminate the possibility that actinobacteria colonies were simply being isolated from plants that contained more pollen. No significant difference was seen in the number of actinobacterial colonies isolated from plants with more pollen per collection than plants with less pollen per collection (Supplementary Figure 2).

### Pollen plating and actinobacterial isolation

200 μL of sterile PBS buffer was aliquoted into to each Eppendorf tube containing collected anthers. Samples were then ground with a sterile pestle, vortexed, and diluted 1:10 with sterile PBS. 50 μL of the dilution was then added to duplicate chitin plates and humic acid-vitamin (HV) plates containing cycloheximide and nystatin to reduce fungal growth (Chevrette et al., 2019). Plates were grown for 1–4 weeks at 30 °C. Plates were checked regularly and colonies with actinobacterial morphology were counted. Actinobacteria colonies were removed from the isolation plates and streaked onto yeast malt extract agar (YMEA) plates, with antifungals, and grown at room temperature before DNA extraction and competition assay.

### Competition assay

Actinobacteria were plated from one-week cultures on solid ISP2 (Difco) media onto the left half of each well of a 12 well plate containing yeast peptone malt extract (YPM) agar. Actinobacteria were grown for 7 days at 30 °C before pathogens were added. Bee and plant pathogens were obtained from the USDA ARS Culture Collection and were grown in liquid yeast, peptone, malt (YPM) culture at 30 °C and 250 rpm for 24 h before plating. Liquid culture pathogens were stamped onto the right side of each well and grown for an additional 5–7 days at 30 °C before determining if the actinobacteria had no, some, or complete inhibition of a given pathogen. Pathogens without a competing actinobacteria and blank wells were included on a separate plate as negative controls. Bioassays were repeated three times, and the scores reported are the average of the three bioassays.

### DNA extraction

DNA was extracted from each actinobacteria strain sample using the Masterpure Yeast DNA Purification Kit. A single colony was taken from cultures grown on ISP2 plates and placed into liquid culture. Strains were inoculated in yeast malt extract broth (YMEB) at 30 °C and 250 rpm for approximately 2 weeks prior to extraction. Strain samples were placed into clean centrifuge tubes and centrifuged for 2 min at 15,000 rpm. Supernatant was discarded and 300 μL of “Yeast Cell Solution” was added to each sample pellet. Samples were vortexed and placed into a 65 °C hot water bath for 15 min. Then, the samples were placed into an ice bucket for 5 min minimum. 150 μL of “MPL Protein Precipitation” was added and then samples were vortexed. Samples were then centrifuged for 10 min at 15,000 rpm and supernatant was transferred to a clean tube. 500 μL of isopropanol was added and tubes were inverted several times. Samples were centrifuged again for 10 min at 15,000 rpm and the supernatant was discarded. Pellets were washed with 0.5 mL of 70% EtOH and then centrifuged briefly to remove remaining EtOH. 35 μL of TE buffer was added to each sample pellet and then stored at 4 °C.

### 16S amplification and sequencing

An initial taxonomic identification was performed by 16S analysis. The 16S rRNA gene was amplified from the isolated genomic DNA via PCR. The PCR reaction contained 1 μL of template DNA, 12.5 μL of EconoTaq, 1 μL of the universal bacterial primers, 27F (5’-AGAGTTTGATCCTGGCTCAG-3′) and 1496R (5’-CGGTTACCTTGTTACGACTT-3′) and 12.5 μL nuclease free water. The thermocycler program consisted of an initial 3 min denaturation step at 95 °C, followed by annealing at 58 °C for 3 min, followed by 35 cycles of 10 s at 96 °C and 2 min at 72 °C, and a final extension at 72 °C for 7 min. Amplified 16S rDNA was sequenced by Sanger sequencing using the universal bacterial primer 27F.

### 16S phylogeny

The sequenced 16S sequences were manually inspected for quality and trimmed using MEGA X (Kumar et al., 2018). A multisequence alignment was created using ClustalW in MEGA X using the 16S sequences and type strain *Streptomyces* sequences collected from NCBI. The multisequence alignment was used to create a Maximum likelihood phylogenetic tree with 100 bootstraps (Supplementary Figure 3).

### Genome sequencing and assembly

Selected strains were sequenced through the University of Wisconsin-Madison Biotechnology Center using an Illumina MiSeq 2 × 150 bp paired-end sequencing. The raw reads were trimmed, corrected and checked for quality with fastp (Chen et al., 2018). Draft genome sequences were assembled using SPADES v3.15.3 (Prjibelski et al., 2020). The genome assembly quality was assessed in KBase using QUAST and CheckM (Supplementary Figure 12). The draft genomes reported in this study can be found on NCBI at the following BioProjectID PRJNA1266655.

### Multi-locus sequencing typing

Multi-Locus Sequence Typing was used to determine a more accurate taxonomic determination for the sequenced strains. AutoMLST2.0 was used with all default parameters to identify the likely taxonomy of each of the sequenced strains (Pourmohsenin et al., 2025). The list of genes used in creating the multilocus sequence alignment is included in Supplementary Figure 4. The AutoMLST2.0 data was visualized with iTOL to generate a phylogenetic tree of the sequenced strains (Letunic and Bork, 2024).

### Genomic analysis

Draft genomes were annotated using RASTtk-v.1.073 in the KBase platform and the annotations of each genome were searched for genetic markers of endophytism as identified in previous publications (Arkin et al., 2018). Genetic markers of endophytism were confirmed using other relevant search tools and databases. AntiSMASH was used to confirm the presence of ionophore BGCs, dbCAN3 was used to confirm the presence of carbohydrate acting enzymes, and BLAST was used to confirm the presence of plant hormone biosynthetic genes (Zheng et al., 2023).

### Biosynthetic gene cluster identification and visualization

Biosynthetic Gene Clusters were identified using AntiSMASH v.7.0 with comparison to the MIBiG Database (Blin et al., 2023; Terlouw et al., 2023). AntiSMASH results were manually curated to look for similar biosynthetic gene clusters between the sequenced draft genomes and other known endophytic *Streptomyces* genomes. Biosynthetic gene clusters of interest were visualized using Clinker through the CAGECAT platform (van den Belt et al., 2023). In Clinker, only the best matches to biosynthetic genes were kept and gene identifications were assigned by performing a BLASTP analysis on one representative of each homologous gene.

### LC-MS/MS

*Streptomyces* isolates were grown on ISP2 solid media for 2 weeks before five agar cores were taken from the edge of the mycelial growth for each plate. The agar cores were macerated in methanol for 2 days and the organic extract was dried *in vacuo*. The extracts were resuspended in methanol and eluted through C18 Sep-Pak filters with methanol to prepare the samples for LC-MS/MS analysis. Extracts were analyzed on a Dionex UPLC system coupled to a ThermoScientific Q-Exactive quadrupole orbitrap mass spectrometer. The UPLC system was 5% methanol in water with 0.1% formic acid for 0.5 min followed by a gradient from 5% methanol to 100% methanol with 0.1% formic acid over 15 min. A 100% methanol wash was held for 2 min before switching back to 5% methanol over 0.5 min and re-equilibrating at 5% methanol for 1 min. The flow rate was 0.35 mL/min. This method was run on a 2.1 × 100 mm, 2.6-micron Phenomenex Kinetex XB-C18 column. The mass spectrometer scanned from 200 to 2,000 *m/z* in positive mode. The mass spectrometry datasets analyzed for this study can be found in MASSive at: ftp://massive-ftp.ucsd.edu/v09/MSV000097971/.

### Metabolomics

The raw data for each isolate was manually inspected and filtered using MzMINE3 followed by the creation of an aligned feature table and quantification table for feature-based molecular networking (Schmid et al., 2023). Feature-based molecular networking was performed through GNPS with a precursor ion mass tolerance of 0.05 Da, a fragment ion mass tolerance of 0.05 Da, a minimum cosine matching score of 0.7, and a minimum of six matched fragment ions (Nothias et al., 2020). GNPS spectral libraries were searched for matches to the submitted MS/MS spectra. Additionally, M + H and M + Na values for suspected compounds were manually searched using MzMine3 and their MS/MS spectra analyzed by both MzMine3 and GNPS.

## Results

### Phylogenetically diverse actinobacteria are isolated from plant and hive pollen

Five replicate samples of pollen were collected from ten different plants (Supplementary Figure 1) along the University of Wisconsin Lakeshore Nature Preserve in April and June. Ten pollen stores were collected from *Apis mellifera* hives at the University of Wisconsin-Madison. A portion of each pollen sample was suspended and vortexed in sterile PBS and spread onto 5 replicate chitin plates. Colonies with actinobacterial morphology were identified and counted for each pollen sample. Averages of actinobacterial-like colonies were then calculated for the five replicate chitin plates. Putative actinobacterial colonies were picked and cultured on ISP2 plates. Actinobacteria were identified from pollen of four of the ten plants studied, while all the hive samples contained actinobacteria-like colonies.

DNA was extracted from the isolated strains and the 16S rRNA gene was amplified and sequenced to make an initial identification of the isolated strains. The 16S rRNA sequences were analyzed by BLAST and compared to the NCBI nucleotide database. Most of the isolates had the greatest percent identity to members of the genus *Streptomyces*; however, several isolates belonged to rare genera of the Actinomycetota. Three isolates had the greatest percent identity to members of *Micromonospora*, one had the greatest percent identity to *Kribella*, and one had the greatest percent identity to *Dactylosporangium* (Supplementary Figure 1). Upon creating a maximum likelihood tree of the *Streptomyces* 16S sequences, there was no discernable phylogenetic pattern for the source of the isolates (plant-isolated vs. hive-isolated). Rather, isolates taken from different plants and pollen stores clustered together on the maximum likelihood tree (Supplementary Figure 3). This demonstrated that actinobacteria from hives are not a unique group of *Streptomyces*, rather they are phylogenetically diverse and cluster with various *Streptomyces* spp. isolated from plants.

**Figure 1:**
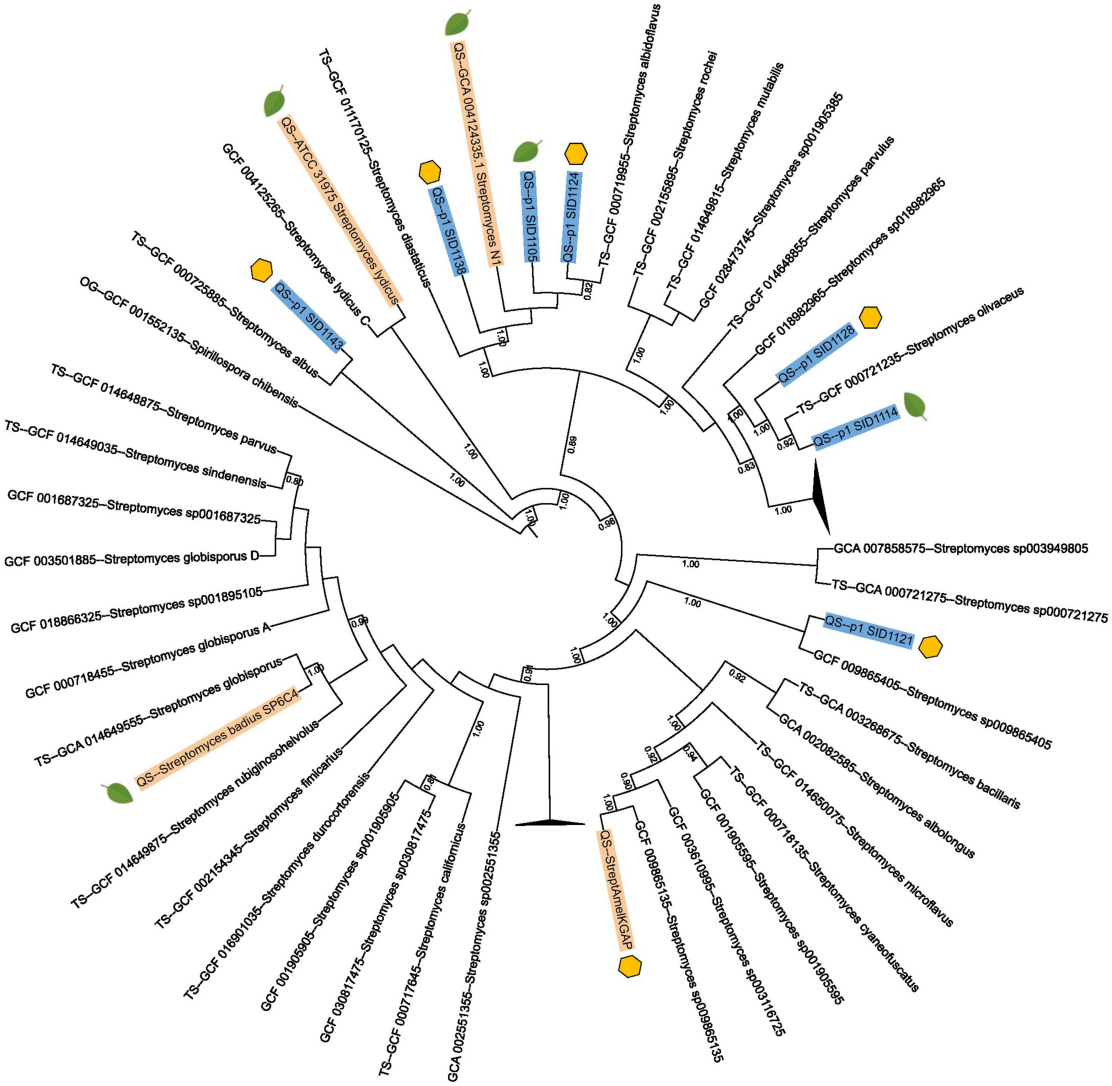
Multi-locus species tree of sequenced *Streptomyces* isolates and previously published *Streptomyces* strains. Strains sequenced in this study are highlighted in blue and previously published *Streptomyces* strains are highlighted in gold. *Streptomyces* type strain genomes from AutoMLST2.0 are also included in the tree. Strains isolated from hive pollen are marked with a yellow hexagon while strains isolated from plant pollen or plant tissues are marked with a green leaf. The numbers listed on branches represent ANI values and only ANI values above 0.7 are shown.

Seven of the *Streptomyces* isolates were selected for full genome sequencing to compare their genomes to the characterized endophytic strains *Streptomyces lydicus* ATCC 3197, *Streptomyces* sp. N1 and *Streptomyces badius* SP6C4 as well as the hive-isolated *Streptomyces* sp. Amel-AP1 (Kim et al., 2019; Worsley et al., 2020; Grubbs et al., 2021). To obtain a more accurate taxonomy for the sequenced strains, a multi-locus species tree was created using AutoMLST2.0 and iTOL. Of the seven strains that were sequenced, six had an average nucleotide identity (ANI) of 97% or greater to known *Streptomyces* type strains. Interestingly, three of the isolates, two from hives (SID1124 and SID1138) and one from a plant (SID1105), had high ANI to each other and to the previously characterized endophyte *Streptomyces* sp. N1. All four of these strains had high ANI to the type strain *Streptomyces albidoflavus*. Another two isolates, one from hives (SID1128) and one from a plant (SID1114), had high ANI to each other and to the type strain *Streptomyces olivaceus* (Figure 1). Both *Streptomyces albidoflavus* and *Streptomyces olivaceus* have been reported from plant and rhizome material and are often investigated for their plant pathogen inhibiting activities (Du et al., 2022; Um et al., 2022). The other two isolates, both from hives, had high ANI to *Streptomyces albus* (SID1143) and *Streptomyces scopuliridis* (SID1121).

### Plant and hive-isolated *Streptomyces* contain endophytic genetic markers

To determine if the isolated strains from both plant pollen and hive pollen originated from plants, the genomes of the isolates and three characterized endophytic strains (*Streptomyces badius* SP6C4, *Streptomyces* sp. N1, and *Streptomyces* sp. ATCC 31975) were analyzed for genetic markers of endophytism. The genomes were analyzed using KBASE, AntiSMASH, dbCAN3, and BLAST to identify ionophore biosynthetic gene clusters (BGCs), genes that encode enzymes that aid in production of the plant hormones auxin, cytokinins, and ethylene, and genes that encode the plant cell wall degrading enzymes endoglucanase and beta-glucosidase (Table 1). Ionophores help bacteria solubilize metals such as iron and allow them to compete for limited inorganic resources in the rhizosphere and plant tissues. Additionally, the solubilized metals complexed with ionophores can be used by plants to gather additional nutrients from the soil and deprive antagonistic microbes of these nutrients (Kloepper et al., 1980). The plant hormones auxin and cytokinins aid in root and shoot lengthening and several other plant developmental stages (Werner and Schmülling, 2009; Gomes and Scortecci, 2021). While ethylene production has a variety of effects, such flower opening, fruit ripening, and adaptation to stress (Gamalero and Glick, 2015), endoglucanase and beta-glucosidase can break down cellulose in plant cell walls and allow endophytic bacteria to consume the released sugars and access plant tissues. Together, these endophytic markers encode genes or groups of genes that would allow a microbe to compete for metals in the rhizosphere and the metal depleted environment of the plant tissue, produce hormones to affect the growth of associated plants, and consume sugars from the cell walls of associated plants. Each of these genetic markers has been described for characterized endophytic *Streptomyces* isolates and other characterized endophytic bacteria (Subramaniam et al., 2020; Worsley et al., 2020).

**Table 1:** Presence or absence of genetic markers of endophytism.

Strain	Isolate Source	Ionophore BGC (#)	Auxin biosynthesis						Ethylene production	Cytokinin Biosynthesis	Endo glucanase	Beta-glucosidase
			EC 2.4.2.18	EC 4.2.1.20	EC 1.2.3.7 EC 1.2.1.3	EC 1.13.12.3	EC 3.5.1.4 EC 3.5.1.-	EC 1.4.3.4 EC 1.4.3.22	EC 3.5.99.7	EC 2.5.1.75	EC 3.2.1.4	EC 3.2.1.21
**1105**	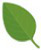	Yes (2)	X	X	X		X	X	X	X	X	X
**1114**	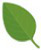	Yes (1)	X	X	X		X			X	X	X
**1121**	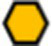	Yes (2)	X	X	X		X			X	X	X
**1124**	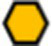	Yes (2)	X	X	X		X	X	X	X	X	X
**1128**	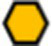	Yes (1)	X	X	X		X			X	X	X
**1138**	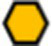	Yes (3)	X	X	X		X	X	X	X	X	X
**1143**	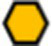	Yes (2)	X	X	X		X	X		X	X	X
Amel AP-1	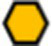	Yes (3)	X	X	X		X	X		X	X	X
SP6C4	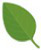	Yes (1)	X	X	X		X			X	X	X
N1	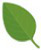	Yes (2)	X	X	X	X	X	X	X	X	X	X
ATCC 31975	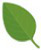	Yes (2)	X	X	X	X	X	X	X	X	X	X

*Streptomyces* strains isolated in this study are bolded. Yellow hexagons represent strains isolated from hive pollen and green leaves represent strains isolated from plant pollen or plant tissues. Enzyme commission numbers are listed for the various genes that were searched in the analysis.

Each analyzed strain contained genes that encode anthranilate phosphoribosyltransferase (EC 2.4.2.18), tryptophan synthase (EC 2.4.1.20), indole-3-acetaldehyde oxidase (EC 1.2.3.7), and acylamidase (EC 3.5.1.4), which are each part of the auxin biosynthetic pathway. Some strains contained additional genes that encode enzymes involved in auxin biosynthesis, such as tryptophan 2-monooxygenase (EC 1.13.12.3) and monoamine oxidase (EC1.4.3.4). Each of the analyzed strains also contained genes that encode tRNA dimethylallyltransferase (EC 2.5.1.75), which is a critical step in the cytokinin biosynthetic pathway, and 1-aminocyclopropane-1-carboxylate deaminase (EC 2.5.1.75), which produces the immediate precursor to ethylene. Additionally, each of the strains contained genes that encode beta-glucosidases (EC 3.2.1.21) and genes that encode endoglucanases (EC 3.2.1.4). All analyzed strains contained BGCs for at least one ionophore with nearly all containing a BGC for desferrioxamine-like hydroxymate siderophores. Several others contained the BGC for coelichelin-like peptidic siderophores in addition to the desferrioxamine-like siderophores (Table 1). The similarity in genetic markers between characterized endophytic *Streptomyces* and hive-isolated *Streptomyces* provides further evidence that the hive-isolated *Streptomyces* may have originated from plants.

### Plant and hive-isolated *Streptomyces* encode similar natural product biosynthetic gene clusters

While analyzing the sequenced genomes using AntiSMASH, it was noted that several of the sequenced strains from both plants and hives encoded similar BGCs in addition to the ionophore BGCs. BGCs for the nonribosomal peptide synthetase/polyketide synthase (NRPS/PKS) hybrid polycyclic tetramate macrolactams (PoTeMs) were present in SID1105, SID1114, SID1124, SID1128, and SID1138. A BGC for the NRPS derived surugamides was identified in SID1138 and fragments of surugamide-like BGCs were identified in SID1105 and SID1124 due to poor assembly of the genomes for those strains. Similarly, fragments of the BGC that encodes the PKS-derived lobophorins were identified in SID1128 and SID1114. The BGC for the hydroxymate siderophore desferrioxamine was also identified in all strains. These four BGCs have been found in other previously characterized endophytic strains, suggesting that these BGCs are broadly found in endophytic *Streptomyces* and likely important for surviving in this niche (Worsley et al., 2020; Um et al., 2022; Chen et al., 2025).

**Figure 2:**
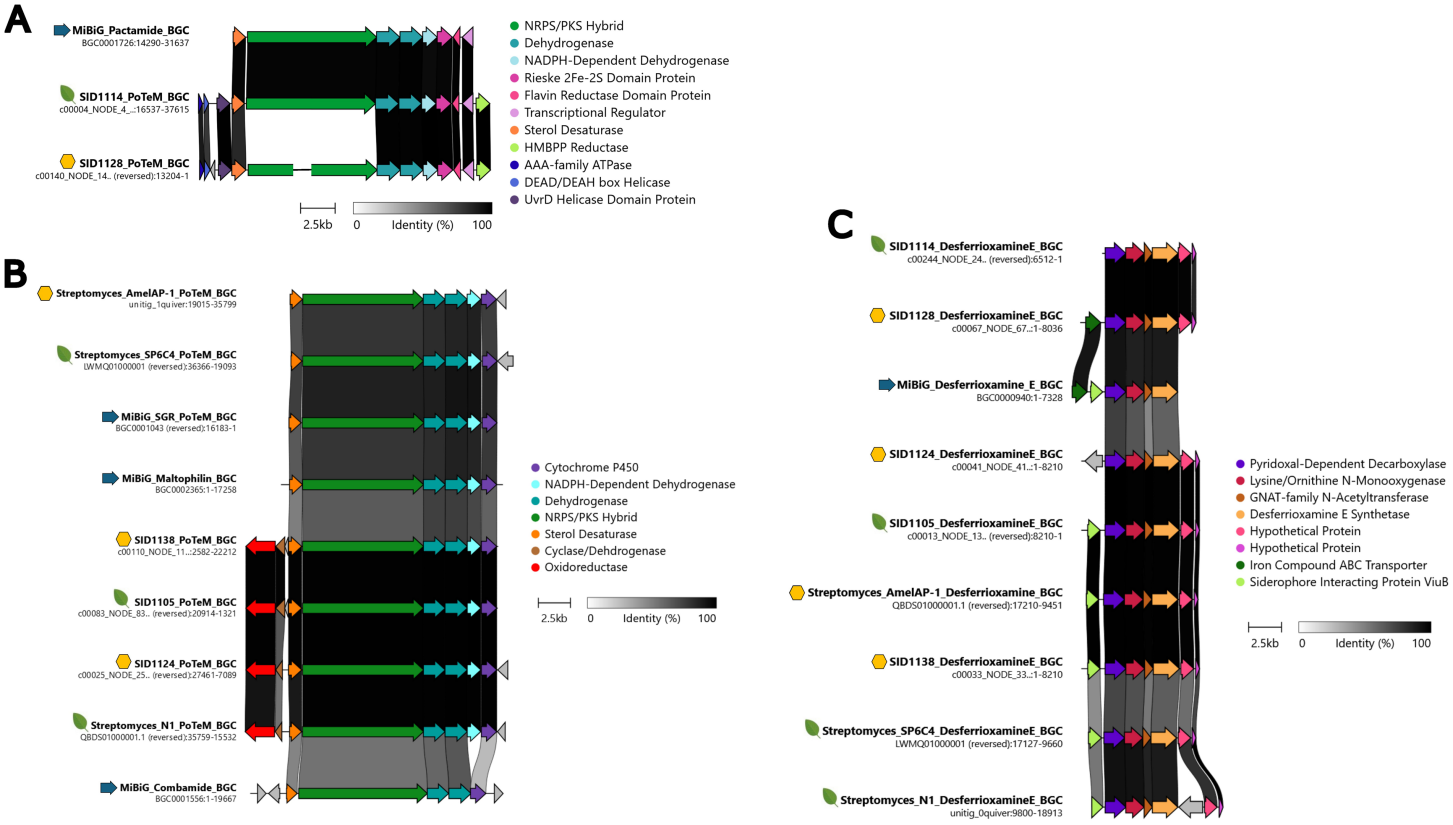
Clinker comparisons of the BGCs identified via AntiSMASH in the sequenced *Streptomyces* isolates. **(A)** Pactamide-like PoTeM BGCs for SID1114 and SID1128, **(B)** Maltophilin-like PoTeM BGCs for SID1138, SID1105, and SID1124, **(C)** Desferrioxamine E-like BGCs for SID1114, SID1128, SID1124, and SID1105, and SID1138. Yellow hexagons represent BGCs from hive pollen isolated strains, green leaves represent BGCs from plant pollen- or plant tissue-isolated strains, and blue arrows represent MiBiG database BGCs. BGCs were arranged to show the highest percent identity between BGCs, represented by the dark bars between genes.

PoTeMs are a chemically diverse group of macrolactams with several reported activities ranging from antibacterial to antifungal activity (Ding et al., 2021). The three *Streptomyces albidoflavus* strains, SID1105, SID1124, and SID1138, all contained PoTeM BGCs that were more similar to each other than to other characterized PoTeM BGCs, while SID1114 and SID1128 contained a PoTeM BGC that had high similarity to the known pactamide and combamide BGCs (Figures 2A,B). The structures of PoTeMs can be classified as containing a 5 membered ring system, a 5–5-6 membered ring system, or a 5-6-5 membered ring system. These different structural motifs loosely correlate with the number of dehydrogenases present in the BGCs of the producing strain (Blodgett et al., 2010; Quezada et al., 2017). Based on the number of dehydrogenases in the BGCs and a phylogenetic analysis of the NRPS/PKS hybrid enzyme, it is likely that the PoTeMs produced by SID1105, SID1124, and SID1138 are 5–5-6 membered PoTeMs, while SID1114 and SID1128 likely produce 5 membered ring or 5–5 membered ring PoTeMs like pactamides (Figure 2; Supplementary Figure 5). Surugamides are a group of previously characterized cyclic peptides with anti-protease activity and some mild antifungal activity (Takada et al., 2013; Fazal et al., 2020). Despite fragmented genome assemblies, fragments of the surugamide BGCs were able to be identified for the three *Streptomyces albidoflavus* strains (Supplementary Figure 6). All BGCs had very high similarity to the surugamide BGC identified in *Streptomyces* sp. N1, a previously characterized endophyte. The poor assembly of the genomes for SID1114 and SID1128 made it difficult to identify larger BGCs, like the lobophorin encoding PKS. However, genes with high similarity to characteristic lobophorin biosynthesis genes, such as the glycosyltransferases and thioesterase, suggested the presence of a highly fragmented lobophorin encoding BGC (Supplementary Figure 7). Lobophorins have been reported from both endophytic and stingless bee associated *Streptomyces* spp. and have been shown to have antimicrobial properties (Rodríguez-Hernández et al., 2019; Um et al., 2022; Chen et al., 2025). The BGC for the siderophore desferrioxamine was found in all sequenced genomes (Figure 2C).

### LC-MS/MS identifies production of predicted natural products

**Figure 3:**
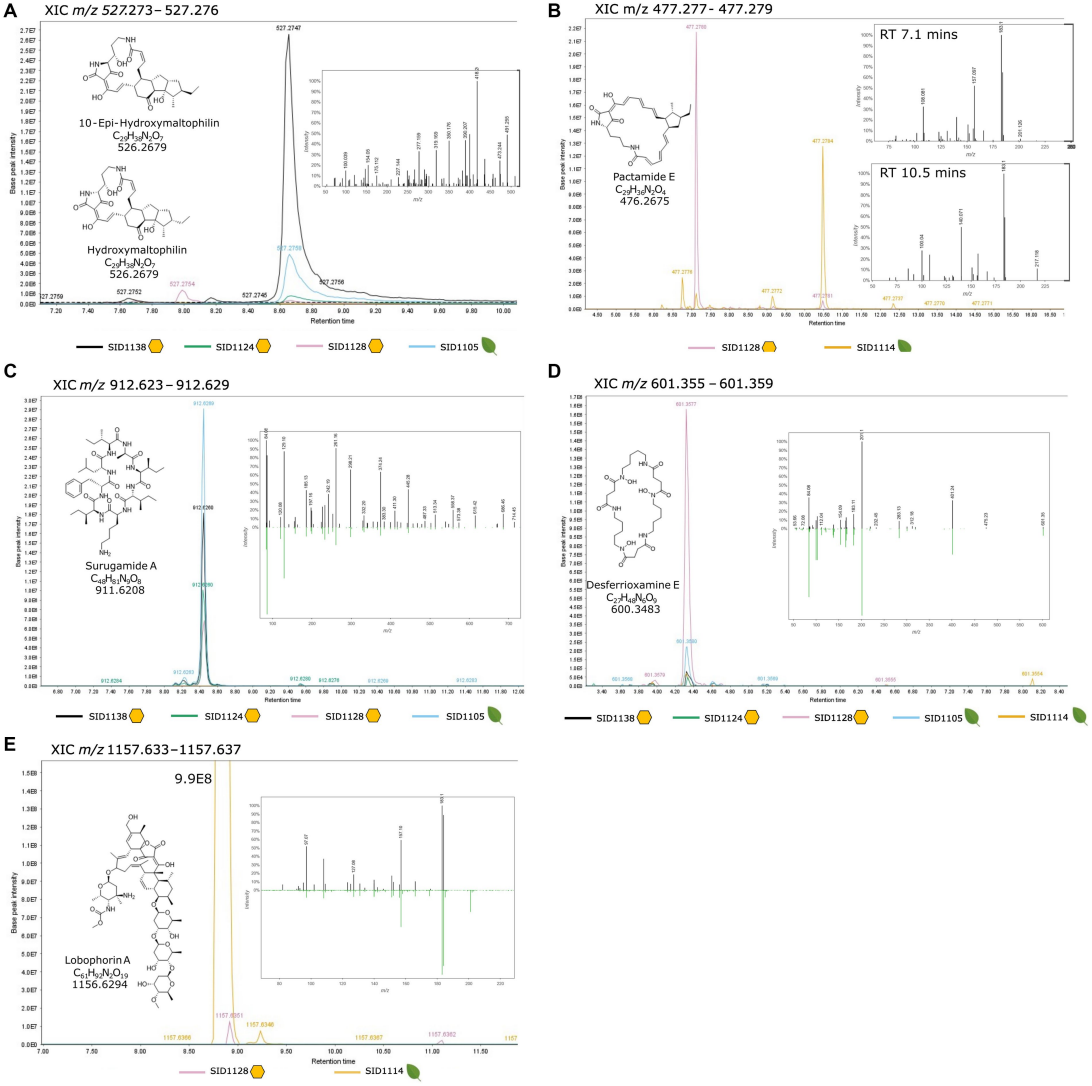
Extracted ion chromatograms from MzMine3 **(A)** Maltophilin-like PoTeMs, **(B)** Pactamide-like PoTeMs, **(C)** Surugamide A, **(D)** Desferrioxamine E, **(E)** Lobophorin A. Each isolated strain is represented by a different colored trace in the XIC and the range for the XIC is given at the top of each section. Yellow hexagons represent hive pollen-isolated strains and green leaves represent plant pollen- or plant tissue-isolated strains. The chemical structure, molecular formula and monoisotopic mass for each compound is listed within the XIC. The inset spectrum is the MS/MS spectrum for the listed *m/z* and is provided as a mirror plot when a direct comparison to a GNPS standard (downward green peaks) was available.

To confirm the production of the BGC-predicted molecules, small organic extracts were created by taking agar cores from each strain growing on solid ISP2 media and macerating the agar cores in methanol overnight. The extracts were dried *in vacuo* and analyzed by LC-MS/MS. The resulting LCMS/MS data was analyzed with MzMine3 and GNPS Feature-Based Molecular Networking. A small molecular networking subnetwork of *m/z* values that matched known PoTeMs was identified for the three *Streptomyces albidoflavus* strains, SID1105, SID1124, and SID1138 (Supplementary Figure 8). PoTeMs are chemically diverse and exist as many constitutional isomers, which complicates the identification via mass spectrometry. However, based on the analysis of the core NRPS/PKS hybrid gene and number of dehydrogenases in the BGCs, it is likely that the PoTeMs produced by SID1105, SID1124, and SID1138 are structurally similar to maltophilins (Supplementary Figure 5). We identified *m/z* values that match the [M + H]^+^ of hydroxymaltophilin and 10-epi hydroxymaltphilin (527.2758 [M + H]^+^) (Figure 3A), frontalamide B (509.2648 [M + H]^+^), and maltophilin and 10-epi maltophilin (511.2808 [M + H]^+^) (Supplementary Figure 8). These PoTeMs belong to the 5-5-6 membered ring class of PoTeMs. The PoTeM BGC for SID1114 and SID1128 was distinct from the BGCs in the other isolated strains with higher similarity to the pactamide BGC. A match for the m/z for pactamide E (477.2754 [M + H]^+^) was identified in the extract of SID1114 and SID1128 (Figure 3B). Pactamide E is a 5 membered ring class of PoTeM. A molecular networking subnetwork of m/z values was also identified that matched the known surugamides A-E and G in each of the strains predicted to produce these cyclic peptides (Supplementary Figure 9). Surugamides A-E were also detected in SID1128 despite no obvious BGC being detected in the highly fragmented genome of SID1128. Surugamides B-E also exist as constitutional isomers with varying patterns of the amino acids leucine and isoleucine within the cyclic peptide. Three unique retention times with the same *m/z* in the subnetwork suggested the presence of three of the four conformational isomers. Surugamide A (912.6287 [M + H]^+^), surugamides B-E (898.6131 [M + H]^+^), and surugamide G (884.6052 [M + H]^+^) were able to be further confirmed by a comparison of their MS/MS fragmentation patterns to library standards in GNPS (Figure 3C and Supplementary Figure 10). Lobophorin A (1157.6373 [M + H]^+^) was identified by manually searching the LC-MS/MS data in MzMine3 and then comparing the MS/MS fragmentation patterns of the parent ion to the library standard in GNPS (Figure 3E). Lobophorin A was highly produced in SID1114 and produced in only small amounts in SID1128. Other lobophorin analogs, lobophorin B (1209.5921 [+H]^+^) and lobophorin H (1185.5959 [M + H]^+^), were found to be produced by SID1128 (Supplementary Figure 11). The siderophore desferrioxamine E (601.3562 [M + H]^+^) was identified as a molecular networking subnetwork with m/z matches for SID1105, SID1124, SID1138, SID1128, and SID1114. The MS/MS fragmentation pattern for each strain matched the library standard for desferrioxamine E in GNPS (Figure 3D). Each of these natural products has been reported from endophytic *Streptomyces* in previous publications and the compounds were produced by both hive and plant isolated *Streptomyces*.

### Plant and hive-isolated *Streptomyces* inhibit the growth of plant and bee pathogens

**Figure 4:**
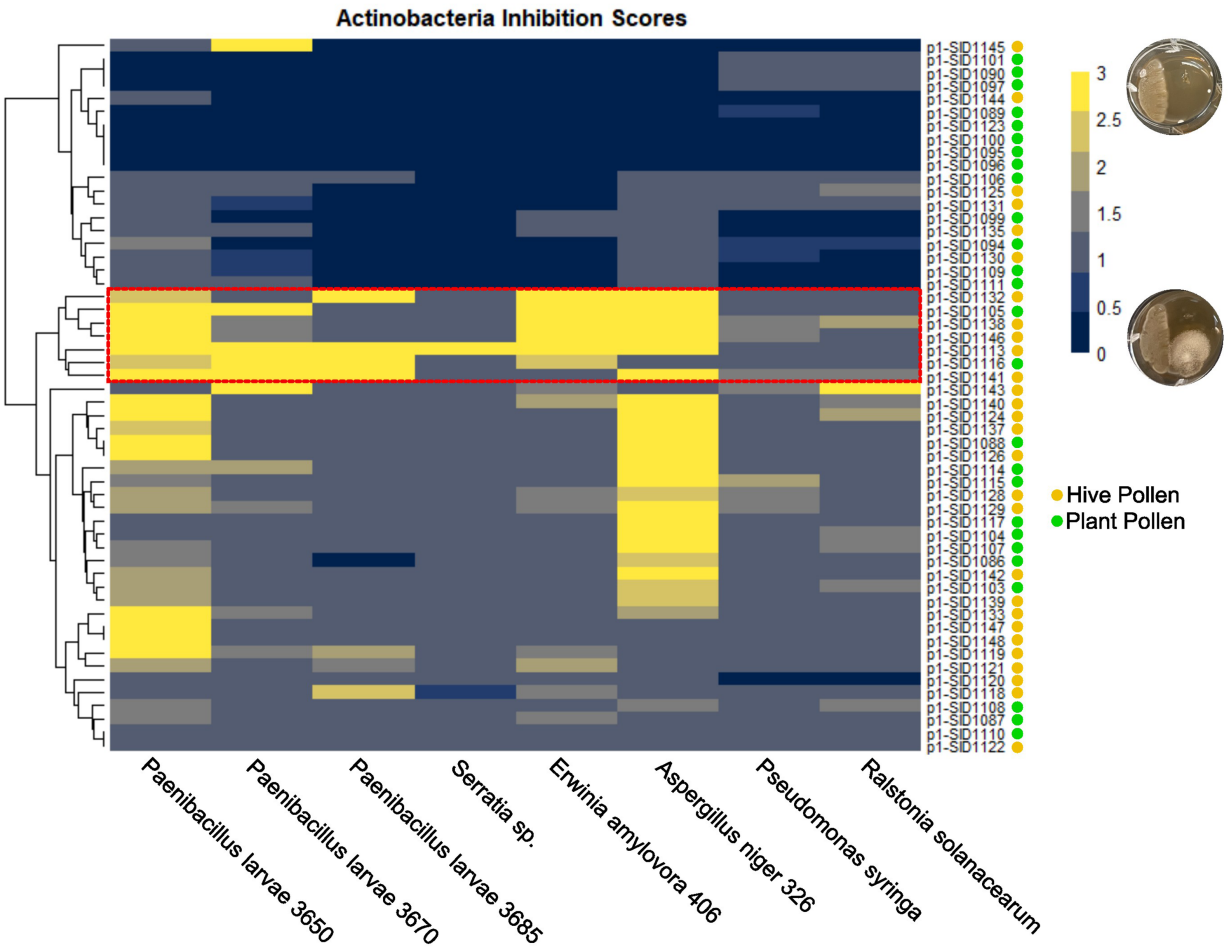
Hierarchically clustered heatmap of isolated actinobacteria inhibition scores against bee pathogens (*P. larvae*, *Serratia* sp., and *A. niger*) and plant pathogens (*E. amylovora*, *P. syringae*, and *R. solanacearum*). Inhibition scores are the average of three independent experiments with yellow representing strong inhibition and dark blue representing no inhibition. Actinobacteria isolates are marked with a yellow circle for hive pollen-isolated strains and a green circle for plant pollen-isolated strains.

Hive isolated *Streptomyces* and endophytic *Streptomyces* have both been shown to inhibit the growth of honey bee pathogens and plant pathogens, respectively. If hive isolated strains are derived from endophytes, it is likely that hive isolates will be able to inhibit both bee and plant pathogens. The isolated strains from both sources were analyzed for their ability to inhibit the honey bee pathogens *Paenibacillus larvae*, *Serratia marcescens*, and *Aspergillus niger* and the plant pathogens *Erwinia amylavora*, *Pseudomonas syringae*, and *Ralstonia solanaceum* in pair-wise competition assays. The isolated strains were cultured on one side of a plate for a week before being challenged with each of the pathogens. Plates were visually inspected and inhibition scores were assigned 0–3 with 0 representing no inhibition, 1 and 2 representing degrees of partial inhibition, and 3 representing complete inhibition of the pathogen (Figure 4). Scores reported in Figure 4 represent the average of three separate pairwise bioassays. The fungal bee pathogen, *Aspergillus niger*, was broadly inhibited with approximately half of the strains having a strong inhibition score of 2–3. The bacterial pathogens were more varied, but isolated strains showed at least some moderate-to-strong inhibition against each bacterial pathogen. When the inhibition scores for all the pathogens were hierarchically clustered, a group of particularly strong inhibitory *Streptomyces* became apparent (outlined in red on the heatmap). This group represented strains derived from both plant and hive sources, including the sequenced strains SID1105 and SID1138. Interestingly, the plant-isolated strongly inhibitory strains originated from two different plant species. Likewise, the hive-isolated strongly inhibitory strains originated from different hive cells. Despite this, two of the inhibitory strains were *Streptomyces* isolates with high average nucleotide identity to *Streptomyces albidoflavus*, a previously described endophytic *Streptomyces* strain. This suggests that, while overall the strains from both isolation sources were taxonomically diverse, specific taxa may be more likely to act as strongly inhibitory isolates that can inhibit bee and plant pathogens. These taxa may represent promising sources of antibiotics for apiculture.

## Discussion

In this study, diverse actinobacteria strains were isolated from plant and hive pollen sources. Actinobacteria were consistently isolated from every hive pollen sample that was studied, whereas actinobacteria isolates were only identified from four of the ten plants that were studied. There are several possible explanations for this discrepancy. It is likely the endophytic association of actinobacteria with plants is not an obligate association, rather it is facultative, with plants associating with certain microbes during specific life stages, seasons, and environmental conditions. Additionally, hive pollen stores are a collection of pollen from several plant species and individuals, increasing the likelihood of having actinobacteria present within a single pollen store. Furthermore, these actinobacteria were phylogenetically diverse with representatives from four different genera: *Streptomyces*, *Micromonospora*, *Kribella*, and *Dactylosporangium*. Hive pollen stores contained multiple phylogenetically diverse actinobacterial isolates, suggesting these isolates came into the hive from different sources as opposed to being unique to a given hive. While there was diversity among the isolates from individual hive pollen stores, several isolates from plants and hives had high ANI and claded together on a maximum likelihood multilocus tree. This suggests that there may be certain actinobacteria such as *Streptomyces albidoflavus* and *Streptomyces olivaceus* that frequently act as endophytes and may be found frequently in both plants and hives.

The similarities in endophytic genetic markers between characterized endophytes and the pollen isolated *Streptomyces* also strongly suggest that hive isolated *Streptomyces* originate as endophytes. The ability to produce ionophores allows *Streptomyces* to sequester inorganic nutrients for both the bacteria and the associated plant (Kloepper et al., 1980). Production of plant hormones and plant hormone precursors, such as auxin, cytokinins, and ethylene, allows *Streptomyces* to interact with and affect the growth and development of associated plants (Werner and Schmülling, 2009; Gamalero and Glick, 2015; Gomes and Scortecci, 2021). Endoglucanases and beta-glucosidase allow *Streptomyces* to liberate simple sugars from the cellulose of plant cell walls and enter plant tissues. While these genetic markers individually may be widespread in actinobacteria from many environmental niches, the combination of all these markers in characterized endophytes reinforces the close association these *Streptomyces* have with plants. Interestingly, isolates from both plants and hives also had similar natural product BGCs to characterized endophytes. Endophytic actinobacteria have been reported to produce PoTeMs, surugamides, and lobophorins (Rodríguez-Hernández et al., 2019; Menegatti et al., 2020; Worsley et al., 2020). These natural products all have various described activities but could be beneficial in competition or communication with other microbes in the rhizosphere, endosphere, or in pollen stores. Inhibition of both plant and honey bee pathogens in pairwise assays further supports this hypothesis. While it is uncertain which natural products were responsible for inhibition of each pathogen, *Streptomyces* likely produce several of these compounds at one time to defend themselves and their access to nutrients in their environment.

Actinobacteria represent promising candidates for defensive microbial symbioses due to their ability to produce many natural products and their ease of vertical transmission via spores. Members of the Actinomycecota have been described in several insect-microbe defensive symbioses; most notably, in symbiosis with fungus farming ants, bee wolves, and southern pine beetles (Currie et al., 1999; Scott et al., 2008; Kroiss et al., 2010). Honey bees represent one of the most economically and agriculturally important insects worldwide and have found their populations repeatedly fluctuating due to a combination of factors. Increased incidence of diseases and parasites and changes in land use towards monoculture crops are two of the drivers recognized to be detrimental to honey bee populations (Oddie and Dahle, 2024). While no evidence of vertically transmitted actinobacterial symbionts was detected in this study, the repeated detection of diverse actinobacteria in bee hives does demonstrate that these bacteria are likely a regular part of the hive microbiota. The similarities between plant and hive isolated *Streptomyces* and characterized endophytic *Streptomyces* strongly suggest that these bacteria originate from plants and further strengthen the close association that honey bees have with the plants around them. Greater diversity in plant populations is likely to have a positive association with the diversity of actinobacterial endophytes available to foraging honey bees. A greater diversity of endophytic *Streptomyces* could provide a greater diversity of beneficial natural products available within plants and hives. Co-culturing *Streptomyces* with relevant hive and plant associated bacteria and fungi may help to identify new beneficial natural products produced in the hive and endophytic environment. Understanding which *Streptomyces* spp. are most likely to act as endophytes and produce beneficial natural products will aid in the development of biological pest and disease control products that protect both plants and pollinators.

## Data Availability

The datasets presented in this study can be found in online repositories. The names of the repository/repositories and accession number(s) can be found in the article/Supplementary material.
