# Supplementary material for: Endophytic Streptomyces from honeybee hives inhibit plant and honeybee pathogens
Source: Front Microbiol. 2025 Sep 30;16:1644842. doi: 10.3389/fmicb.2025.1644842 (PMC12518290; doi:10.3389/fmicb.2025.1644842)
Supplement: Supplementary file 1 [file Table_1.docx]

| **Isolation Source** | **Pollen Collection Date** | **Strain ID** | **Highest BLAST Hit** | **BLAST %ID** |
| --- | --- | --- | --- | --- |
| Blood Root | 4/4/2021 | None |  |  |
| White Trout Lily | 4/11/2021 | p1-SID1086 | Streptomyces sp PL21 | 99.75 |
| White Trout Lily | 4/11/2021 | p1-SID1087 |  |  |
| White Trout Lily | 4/11/2021 | p1-SID1088 | Streptomyces umbrinus | 100 |
| White Trout Lily | 4/11/2021 | p1-SID1089 | Dactylosporangium darangshiense | 97.09 |
| Toothwort | 4/11/2021 | p1-SID1090 | Rhodococcus sp RG-14 | 99.64 |
| Spring Beauty | 4/11/2021 | p1-SID1091 | Sphingomonas faeni | 100 |
| Spring Beauty | 4/11/2021 | p1-SID1092 |  |  |
| Spring Beauty | 4/11/2021 | p1-SID1093 |  |  |
| Twin Leaf | 4/11/2021 | None |  |  |
| Vanilla Sweet Grass | 5/9/2021 | p1-SID1094 |  |  |
| Vanilla Sweet Grass | 5/9/2021 | p1-SID1095 | Micromonospora krabiensis | 99.5 |
| Vanilla Sweet Grass | 5/9/2021 | p1-SID1096 |  |  |
| Vanilla Sweet Grass | 5/9/2021 | p1-SID1097 | Sphingomonas echinoides | 100 |
| Vanilla Sweet Grass | 5/9/2021 | p1-SID1098 | Micromonospora echinospora | 99.86 |
| Vanilla Sweet Grass | 5/9/2021 | p1-SID1099 |  |  |
| Vanilla Sweet Grass | 5/9/2021 | p1-SID1100 |  |  |
| Couchgrass | 6/9/2021 | None |  |  |
| Red Maple | 4/4/2021 | p1-SID1101 |  |  |
| Red Maple | 4/4/2021 | p1-SID1102 |  |  |
| Red Maple | 4/4/2021 | p1-SID1103 | Streptomyces hydrogenans | 99.76 |
| Red Maple | 4/4/2021 | p1-SID1104 | Streptomyces hydrogenans | 99.61 |
| Red Maple | 4/4/2021 | p1-SID1105 | Streptomyces hydrogenans | 99.73 |
| Red Maple | 4/4/2021 | p1-SID1106 |  |  |
| Red Maple | 4/4/2021 | p1-SID1107 | Streptomyces hydrogenans | 99.63 |
| Willow | 4/4/2021 | p1-SID1108 |  |  |
| Willow | 4/4/2021 | p1-SID1109 | Sphingomonas echinoides | 100 |
| Willow | 4/4/2021 | p1-SID1110 |  |  |
| Willow | 4/4/2021 | p1-SID1111 | Streptomyces recifensis | 99.75 |
| Willow | 4/4/2021 | p1-SID1112 | Bacillus pseudomycoides | 99.88 |
| Willow | 4/4/2021 | p1-SID1113 |  |  |
| Willow | 4/4/2021 | p1-SID1114 | Streptomyces sp. VN1 | 99.87 |
| Willow | 4/4/2021 | p1-SID1115 | Streptomyces sp. VN1 | 99.38 |
| Willow | 4/4/2021 | p1-SID1116 |  |  |
| Common Milkweed | 6/20/2021 | p1-SID1117 | Pseudomonas syringae | 99.52 |
| Pollen Store 1 | 9/8/2020 | p1-SID1118 | Streptomyces sp. TCA20005 | 99.86 |
| Pollen Store 1 | 9/8/2020 | p1-SID1119 | Streptomyces sp CC12J | 100 |
| Pollen Store 1 | 9/8/2020 | p1-SID1120 | Streptomyces sp CC12J | 99.83 |
| Pollen Store 1 | 9/8/2020 | p1-SID1121 | Streptomyces sp CC12J | 100 |
| Pollen Store 1 | 9/8/2020 | p1-SID1122 | Kribella sp. PB-83 | 99.45 |
| Pollen Store 1 | 9/8/2020 | p1-SID1123 | Methylobacterium komagatae | 99.87 |
| Pollen Store 2 | 9/8/2020 | p1-SID1124 | Streptomyces hydrogenans | 99.87 |
| Pollen Store 2 | 9/8/2020 | p1-SID1125 | Streptomyces nogalater | 99.73 |
| Pollen Store 2 | 9/8/2020 | p1-SID1126 | Streptomyces albogriseolus | 99.46 |
| Pollen Store 2 | 9/8/2020 | p1-SID1127 |  |  |
| Pollen Store 3 | 9/8/2020 | p1-SID1128 | Streptomyces olivaceus | 98.91 |
| Pollen Store 3 | 9/8/2020 | p1-SID1129 | Streptomyces olivaceus | 99.7 |
| Pollen Store 3 | 9/8/2020 | p1-SID1130 | Micromonospora sp SMLCC3 | 99.55 |
| Pollen Store 4 | 9/8/2020 | p1-SID1131 | Priestia aryabhattai | 100 |
| Pollen Store 5 | 9/8/2020 | p1-SID1132 |  |  |
| Pollen Store 5 | 9/8/2020 | p1-SID1133 |  |  |
| Pollen Store 5 | 9/8/2020 | p1-SID1134 |  |  |
| Pollen Store 5 | 9/8/2020 | p1-SID1135 | Methylobacterium sp YMA35 | 100 |
| Pollen Store 6 | 9/8/2020 | p1-SID1137 |  |  |
| Pollen Store 6 | 9/8/2020 | p1-SID1138 | Streptomyces hydrogenans | 99.75 |
| Pollen Store 7 | 9/8/2020 | p1-SID1139 |  |  |
| Pollen Store 7 | 9/8/2020 | p1-SID1140 | Streptomyces hydrogenans | 99.54 |
| Pollen Store 7 | 9/8/2020 | p1-SID1141 |  |  |
| Pollen Store 7 | 9/8/2020 | p1-SID1142 | Streptomyces thermocarboxydus | 99.29 |
| Pollen Store 7 | 9/8/2020 | p1-SID1143 | Streptomyces albus | 99.74 |
| Pollen Store 8 | 9/8/2020 | p1-SID1144 |  |  |
| Pollen Store 9 | 9/8/2020 | p1-SID1145 |  |  |
| Pollen Store 9 | 9/8/2020 | p1-SID1146 |  |  |
| Pollen Store 9 | 9/8/2020 | p1-SID1147 |  |  |
| Pollen Store 10 | 9/8/2020 | p1-SID1148 | Bacillus thuringiensis | 100 |
| Pollen Store 10 | 9/8/2020 | p1-SID1149 | Bacillus thuringiensis | 100 |

Supplemental Figure 1: Source of bacterial isolates and initial 16S-based taxonomic ID based on comparison to NCBI via BLAST. Isolates without a taxonomic ID did not have DNA extracted or the 16S sequence did not amplify during PCR. Isolation sources with no isolates listed had no actinobacteria-like isolates identified.

| **Pollen Source** | **Pollen Grains/Isolation Plate** | **Total Actinobacteria-like Colonies** |
| --- | --- | --- |
| Blood Root | 370 | 0 |
| White Trout Lily | 620 | 4 |
| Cutleaved Toothwort | 680 | 1 |
| Virginia Spring Beauty | 380 | 3 |
| Twinleaf | 6,000 | 0 |
| Vanilla Sweet Grass | 290 | 7 |
| Couchgrass | 650 | 1 |
| Red Maple | 1,740 | 7 |
| Willow | 5,100 | 9 |
| Common Milkweed | 1,420 | 0 |
| Pollen Store 1 | 41,000 | 6 |
| Pollen Store 2 | - | 4 |
| Pollen Store 3 | - | 3 |
| Pollen Store 4 | - | 1 |
| Pollen Store 5 | - | 4 |
| Pollen Store 6 | - | 2 |
| Pollen Store 7 | - | 5 |
| Pollen Store 8 | - | 1 |
| Pollen Store 9 | - | 3 |
| Pollen Store 10 | - | 2 |

Supplemental Figure 2: Pollen grains/plate with identified actinobacteria-like colonies for each collection specimen. The pollen from one of the five replicate collections for each plant was quantified. The pollen from a single pollen store was quantified, with the others assumed to be of a similar magnitude.


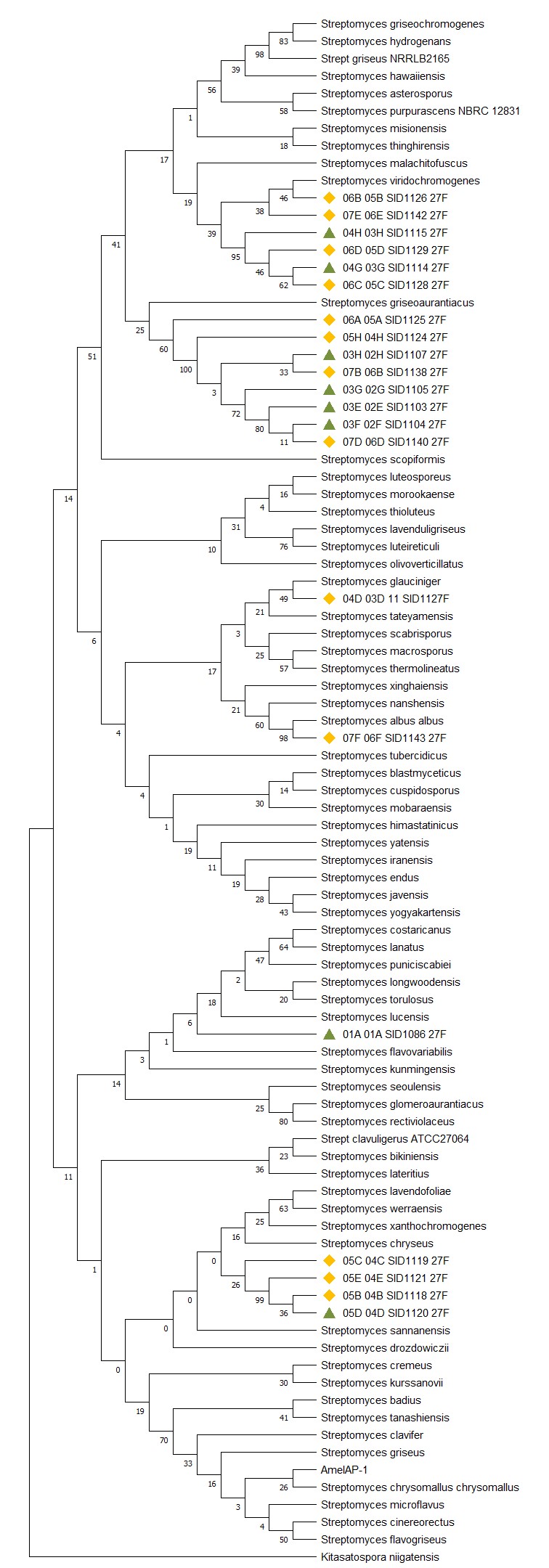


Supplemental Figure 3: Maximum likelihood tree of *Streptomyces* spp. isolates’ 16S sequences. Yellow diamonds represent isolates from hive pollen stores while green triangles represent isolates from plant pollen. Bootstrap values are shown next to branches.

| **Gene ID** | **Description** |
| --- | --- |
| TIGR03654.1 | 50S ribosomal subunit protein L6 |
| TIGR00447.2 | Peptidyl-tRNA hydrolase |
| TIGR00168.2 | Translation initiation factor IF-3 |
| NF004131.0 | 50S ribosomal subunit protein L25 |
| TIGR00096.1 | 30S Ribosomal RNA methyltransferase I |
| TIGR01011.1 | 30S Ribosomal subunit protein S2 |
| NF001616.1 | DNA-directed RNA polymerase subunit beta |
| TIGR01051.1 | Type I DNA topoisomerase |
| NF001810.0 | Elongation Factor P |
| NF003211.1 | Glycine tRNA ligase |
| TIGR00478.1 | TlyA family rRNA (cytidine-2'-O)-methyltransferase |
| TIGR02273.1 | Ribosome maturation factor RimM |
| TIGR00116.1 | Translation elongation factor Ts |
| TIGR01978.1 | Fe-S cluster assembly ATPase SufC |
| NF003828.1 | Rnase adapter RapZ |
| TIGR03625.1 | 50S ribosomal subunit protein L3 |
| TIGR00059.1 | 50S ribisomal subunit protein L17 |
| TIGR01171.1 | 50S ribosomal subunit protein L2 |
| NF010009.0 | DNA integrity scanning diadenylate cyclase DisA |
| TIGR01394.1 | Translation GTPase TypA |

Supplemental Figure 4: List of genes used in AutoMLST2.0 multi-locus sequence alignment


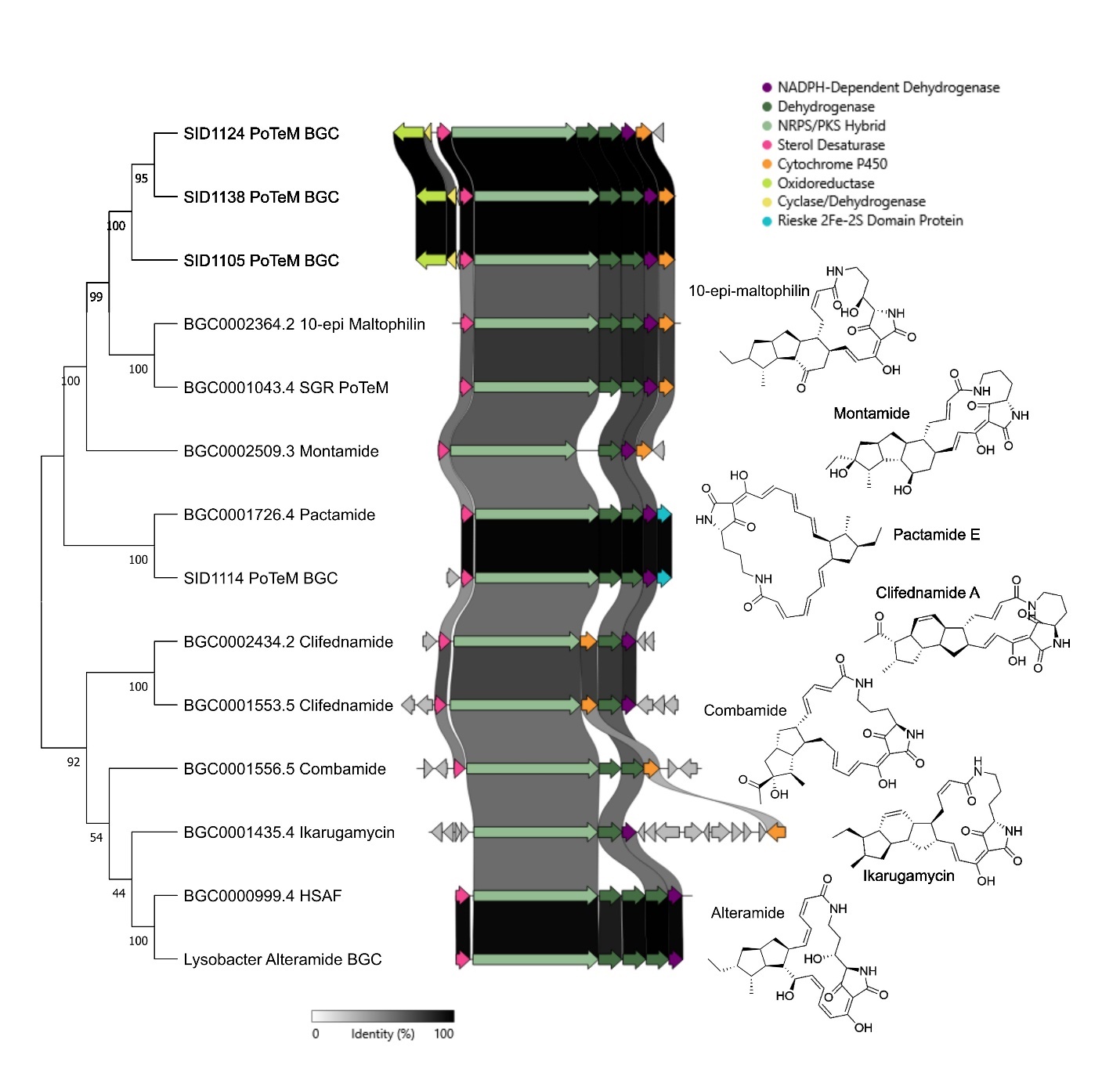


Supplemental Figure 5: Maximum likelihood phylogenetic tree of polycyclic tetramate macrolactam NRPS/PKS hybrid protein sequences with 200 bootstrap replicates. Full BGC similarity shown by Clinker BGC comparison plot with darker lines representing higher percent identity between connected genes. The number of dehydrogenases roughly corresponds to the connected ring structure of the corresponding compounds. Dehydrogenases are shown in dark green.


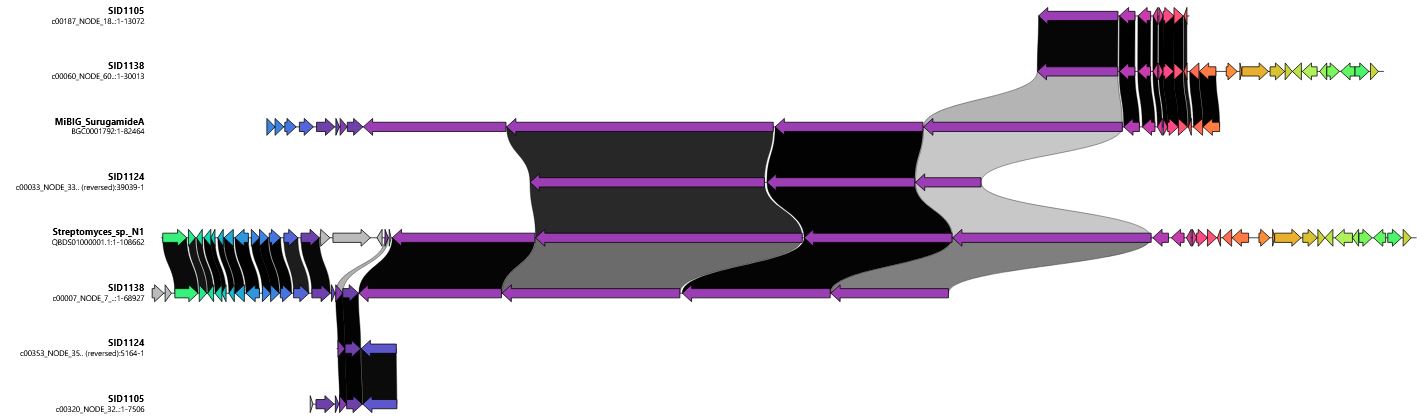


Supplemental Figure 6: Clinker BGC comparison plot of surugamide BGC fragments identified in SID1105, SID1124, and SID1138. Similarly colored genes are homologous and darker lines between genes represent greater percent identity. NRPS genes identified by AntiSMASH are greatly fragmented due to the quality of the sequenced genomes. The surugamide BGC for SID1128 was not detected by antiSMASH, but the molecules were detected by LC-MS/MS. BGCs for surugamide A from the MiBIG database and from the genome of Streptomyces sp. N1 are included to show the full surugamide BGC.


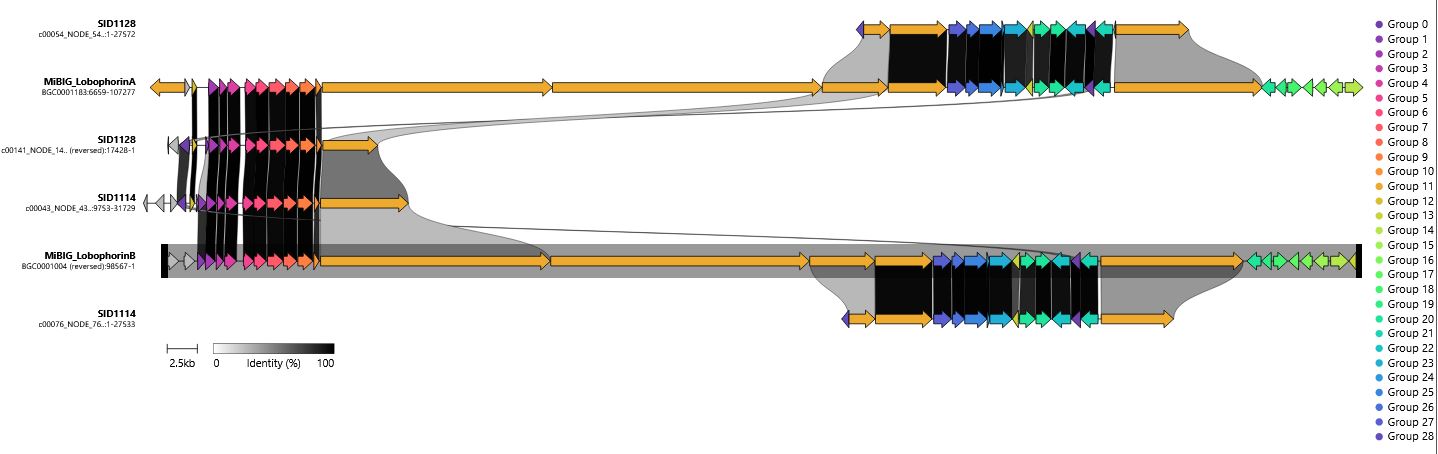


Supplemental Figure 7: Clinker BGC comparison plot of lobophorin BGC fragments identified in SID1114 and SID1128. Similarly colored genes are homologous and darker lines between genes represent greater percent identity. PKS genes identified by AntiSMASH are greatly fragmented due to the quality of the sequenced genomes. BGCs for Lobophorin A and B from the MiBIG database are included to show the full lobophorin BGCs.


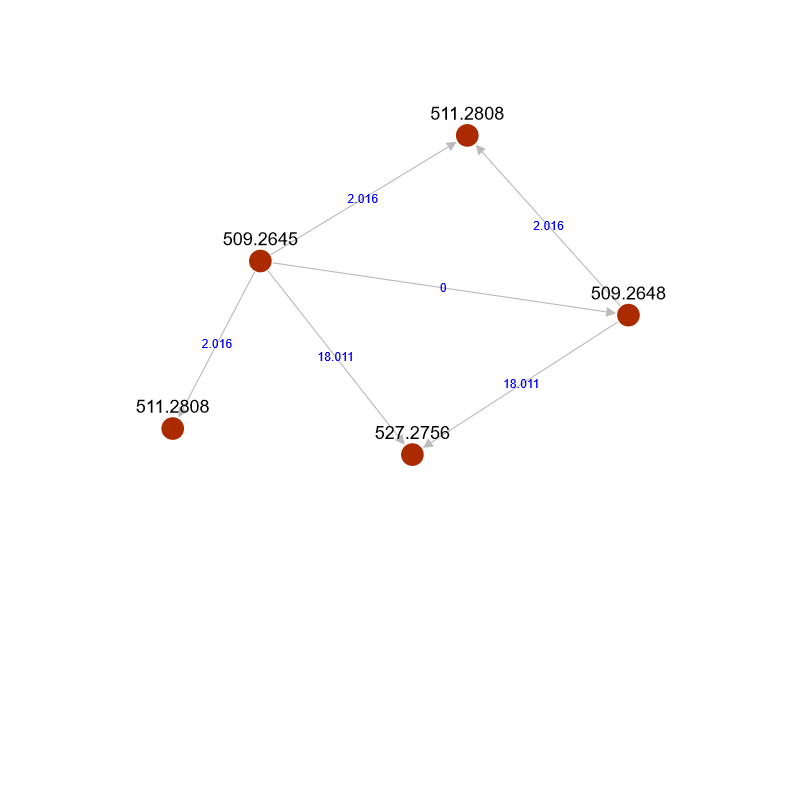


Supplemental Figure 8: Molecular networking subnetwork with *m/z* values that match [M+H]^+^ values of known PoTeMs, frontalamide B (509.2465) maltophilin (511.2803), 10-epi-maltophillin (511.2803), 16-hydroxymaltophilin (527.2752), and 10-epi-hydroxymaltophilin (527.2752). Nodes are labeled with precursor *m/z* values and edges are labeled with Δ*m/z*.


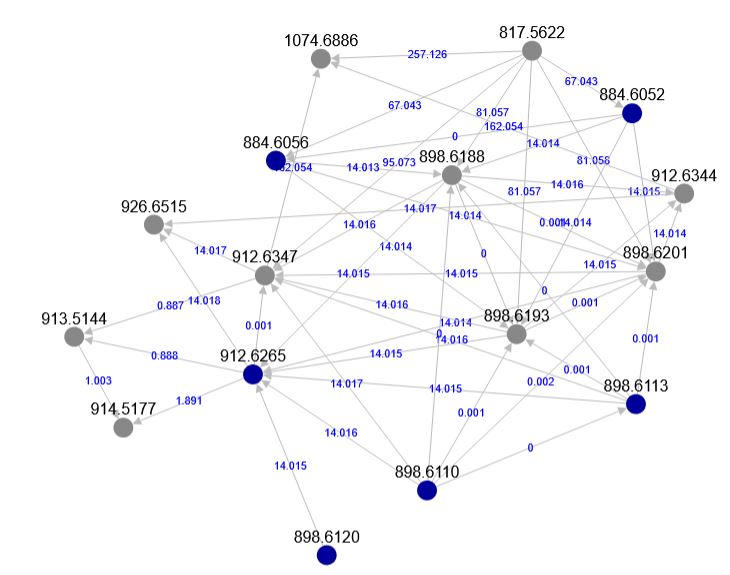


Supplemental Figure 9: Molecular networking subnetwork with *m/z* values that match [M+H]^+^ values of known surugamides: Surugamide A (912.6281), Surugamide B, C, D, E (898.6125), and Surugamide G (884.5968). Nodes are labeled with precursor *m/z* values and edges are labeled with Δ*m/z*. Blue nodes represent MS/MS matches to known surugamides in the GNPS spectral database.


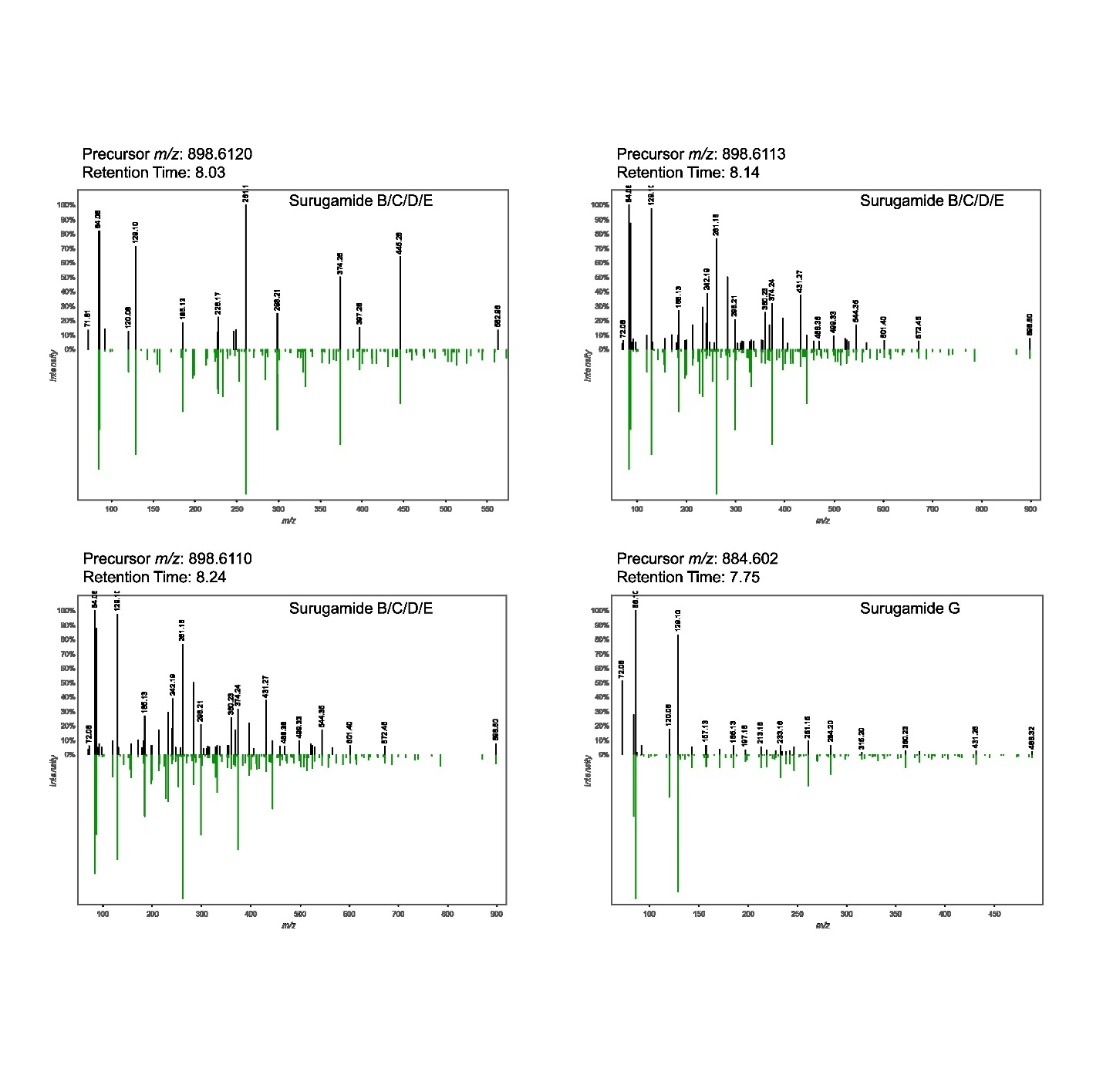


Supplemental Figure 10: GNPS MS/MS matches of surugamides B/C/D/E and Surugamide G. The GNPS library spectrum is in green on the bottom of each mirror spectrum while the query spectrum is on the top.


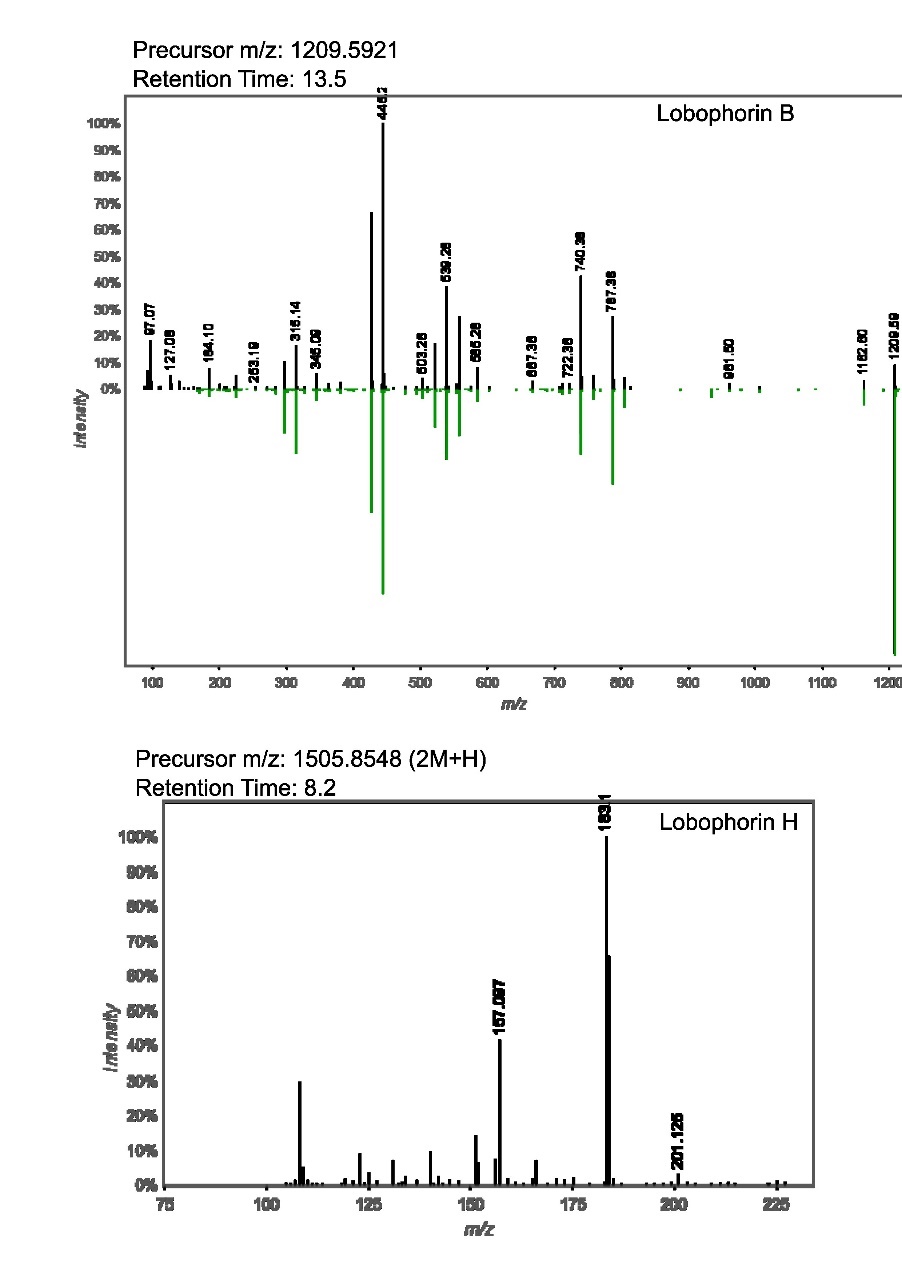


Supplemental Figure 11: GNPS MS/MS matches of lobophorin B and MS/MS spectrum of lobophorin H. The GNPS library spectrum is in green on the bottom of the mirror spectrum while the query spectrum is on the top.

| Genome Assembly Statistics | SID1105 | SID1114 | SID1121 | SID1124 | SID1128 | SID1138 | SID1143 | AmelAP1 |
| --- | --- | --- | --- | --- | --- | --- | --- | --- |
| # contigs | 783 | 768 | 389 | 537 | 1093 | 490 | 271 | 4 |
| Largest contig (bp) | 58890 | 77940 | 171343 | 90460 | 119870 | 95600 | 243100 | 5285582 |
| Total length (bp) | 6995100 | 8244448 | 8169422 | 7015009 | 9221834 | 7186627 | 7779275 | 7783376 |
| N50 | 15852 | 21231 | 38123 | 23543 | 15608 | 26778 | 55336 | 5285582 |
| N75 | 9012 | 10269 | 19933 | 12407 | 7810 | 14042 | 31489 | 2417120 |
| L50 | 138 | 118 | 67 | 93 | 174 | 81 | 43 | 1 |
| L75 | 284 | 261 | 140 | 197 | 383 | 173 | 89 | 2 |
| GC (%) | 73.36 | 72.49 | 71.4 | 73.47 | 72.15 | 73.37 | 72.78 | 71.69 |
| # N's | 0 | 0 | 0 | 0 | 0 | 0 | 0 | 0 |
| CheckM Completeness | 99.03 | 100 | 99.64 | 99.46 | 99.79 | 99.46 | 99.64 | 99.91 |
| CheckM Contamination | 0.79 | 0.43 | 2.72 | 0.57 | 1.17 | 0.57 | 1.25 | 1.26 |

Supplemental Figure 12: Genome assembly quality statistics determined in KBase by QUAST and CheckM.
